# Aboveground biomass estimation at different scales for subtropical forests in China

**DOI:** 10.1186/s40529-017-0199-1

**Published:** 2017-11-09

**Authors:** Shunlei Peng, Nianpeng He, Guirui Yu, Qiufeng Wang

**Affiliations:** 1 Key Laboratory of Ecosystem Network Observation and Modeling, Institute of Geographic Sciences and Natural Resources Research, CAS, Beijing, 100101 People’s Republic of China; 2 Key Laboratory of Ecological Restoration in the Hilly Area, Pingdingshan University, Pingdingshan, 467000 Henan People’s Republic of China; 3 College of Resources and Environment, University of Chinese Academy of Sciences, Beijing, 100049 People’s Republic of China

**Keywords:** Aboveground biomass, Dummy variable model, Wood density, Scale, Allometric equation

## Abstract

**Background:**

The accurate estimation of forest biomass at different scales is the critical step in the assessment of forest carbon stocks. We used three models at increasing scales: allometric model at ecoregional scale (model 1), dummy variable allometric model at both ecoregion and regional scales (model 2), and allometric model at regional scale (model 3) to estimate the aboveground biomass of six subtropical forests in China. Furthermore, we also tested whether wood density can improve the accuracy of the allometric model at regional scale.

**Results:**

Aboveground biomass estimates for six subtropical forests were significantly affected by the ecoregions (*p* < 0.05). Model 1 and model 2 had good fitness with higher values of *R* ^2^, lower *RSE* (residual standard error) and *MPSE* (mean percent standard error) than model 3. The values of *MPSE* for model 1, model 2, and model 3 ranged from 2.79 to 30.40%, 5.15 to 40.94%, and 13.25 to 80.81% at ecoregion scale, respectively. At regional scale, *MPSE* of model 2 was very similar to that of model 1, and was less than model 3. New allometric models with wood density had greater *R* ^2^, lower *RSE* and *MPSE* than the traditional allometric models without wood density variable for six subtropical forests at regional scale.

**Conclusion:**

The dummy variable allometric models have better performances to estimate aboveground biomass for six subtropical forests in China, which provided an effective approach to improve the compatibility of forest biomass estimations from different scales. New allometric models with wood density substantially improved accuracies of aboveground biomass estimation for subtropical forests at regional scale.

**Electronic supplementary material:**

The online version of this article (10.1186/s40529-017-0199-1) contains supplementary material, which is available to authorized users.

## Background

Carbon (C) sequestration and accumulation in forests as aboveground biomass (AGB) is important for mitigating climate change. The estimation of forest biomass at a range of scales has been recognized as one of the most critical steps in the assessment of forest C stocks (Montagu et al. 2005; Tomppo et al. 2010). Tropical and subtropical forests have been reported to account for more than 40% of the global gross primary production (GPP) and net primary production (NPP) (Zhou et al. 2006; Beer et al. 2010; Pan et al. 2011). Long-term eddy covariance observations demonstrate that average net ecosystem production (NEP) of East Asian subtropical forests is 362 g C m^−2^ year^−1^, greater than that of Asian tropical and temperate forests, and also higher than that of forests at the same latitude in North America, Europe and Africa (Yu et al. 2014). Subtropical forest biome in China covers 2.5 × 10^6^ km^2^, occupies about 25% of the total forest area in China (Wu 1995), and plays critical role in C sink and climate change regulating (Zhou et al. 2006; Tan et al. 2011; Yu et al. 2014). However, C budgets of these forests remain uncertain resulted in limited number of inventory plot biomass data and accuracies of allometric equations for estimating AGB of forests in subtropical region (Zhang et al. 2005; Xu et al. 2015; Xiang et al. 2016). Thus, developing allometric equations for subtropical forests is essential for accurate estimating C sequestration in subtropical region (Zaehle et al. 2006; Hudiburg et al. 2009).

Field inventory methods (e.g., harvest method, allomatric modeling, and biomass expansion factor methods) are often used to estimate forest biomass at local and regional scales (Brown et al. 1989; Fang et al. 2001; Wang 2006; Pajtik et al. 2008; Williams et al. 2012). Remote sensing methods can provide spatial information on AGB at large scales, but this method still linked with the relationship between remote sensing dataset and field inventory AGB dataset (Drake et al. 2003; Su et al. 2016). Thus, many scientists gave efforts to improve the tree allometric models at single tree, plot, regional, national, or even worldwide scales, using easily measured dimensional variables, such as diameter at breast high (*DBH*) and tree height (*H*) (Brown et al. 1989; Ter-Mikaelian and Korzukhin 1997; Chave et al. 2005; Návar 2009; Genet et al. 2011). However, different models may lead to greatly variation of biomass estimation because of difference in climatic conditions, site quality, and forest types (Muukkonen 2007; Fu et al. 2017). Therefore, sampling at different scales and creating general biomass model were very important to reduce the uncertainty of applying different models (Chave et al. 2014). Some studies have advanced the possibility of generalizing allometric equations across regional boundaries (Návar et al. 2013; Paul et al. 2013; Chave et al. 2014). Zeng et al. (2011) used dummy variable model to develop generalized biomass model of *Pinus massoniana* at regional scale in south China, and indicated dummy model had good performance. However, few study has compared the accuracy of these allometric equations for forest biomass estimations from site to regional scales (Návar et al. 2013). Moreover, allometric models at different scales for other main subtropical forests in china, such as evergreen broadleaf forest, deciduous broadleaf forest, and mixed forests were very lack (Xu et al. 2015; Xiang et al. 2016). Some studies indicate that wood density variable can greatly improve accuracies of biomass model for AGB estimates in tropical forests and subtropical evergreen broadleaved forest (Baker et al. 2004; Chave et al. 2005, 2014; Goodman et al. 2014; Xu et al. 2015). Chave et al. (2014) successfully developed the universal allometric model for tropical forests with wood density based on global database, which were widely used for AGB estimation in tropical forests. However, the performance of the allometric model with wood density was worth further testing in subtropical forests. Therefore, the development of generalized biomass allometric model at different scales was urgent to quantify the regional biomass and C storage of subtropical forests.

Subtropical region in China has varied ecological zones and forest types (Chinese Academy of Sciences 2001). It was very necessary to develop general biomass models based on ecological region to improve accuracies of AGB estimation. The main objectives of this study were (1) to develop the allometric models at different scales for aboveground biomass estimation of subtropical forests in China; (2) to assessment the accuracy of the allometric models at different scales for AGB estimates, and (3) to test the performance of the allometric model when wood density variable is available.

## Materials and methods

### The experimental site

**Fig. 1:**
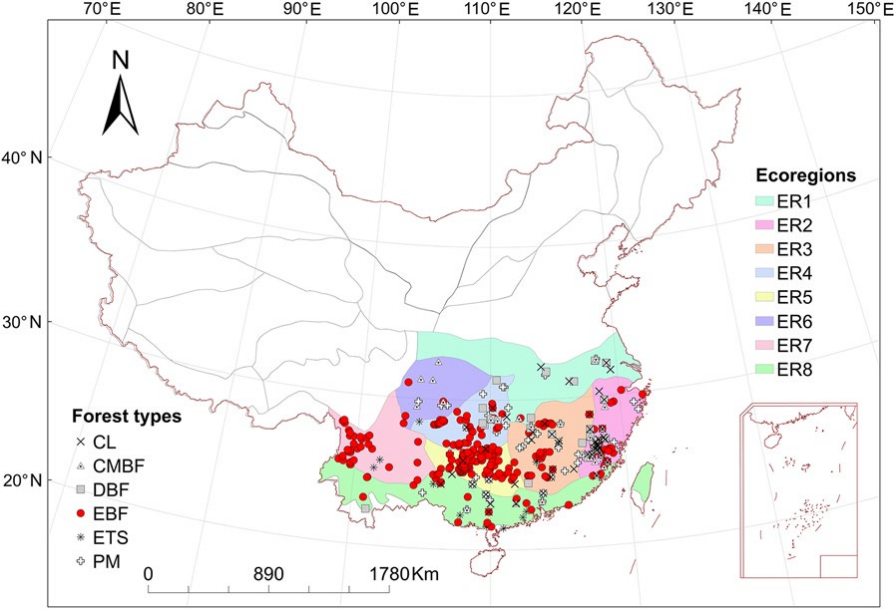
The sampling plot locations for the six forest types in the subtropical region of China. See Table 1 for the abbreviations of the forest types and ecoregions

The study region covered most of the subtropical regions of China (22°–34°N, 98°–123°E) extending across eight ecoregions (Fig. 1). The total forested area is approximately 2.5 × 10^6^ km^2^ in China (Wu 1995). The region was classified eight ecological zones (Table 1; Fig. 1) (Fu et al. 2013). The mean annual precipitation (MAP) ranges from 831 to 1342 mm and the mean annual temperature (MAT) varies from 12.5 to 19.1 °C (Table 1). The primary forests in this region were evergreen broadleaf forests (EBF). Due to long-term anthropogenic disturbances, the current forests were not primitive and were classified into six categories: *Cunninghamia lanceolata* (CL), coniferous mixed broadleaf forest (CMBF), subtropical deciduous broadleaf forest (DBF), evergreen broadleaf forest (EBF), *Eucalyptus* tree species forest (ETS), and *Pinus massoniana* (PM) based on China’s vegetation classification system (Chinese Academy of Sciences 2001).

**Table 1:** Summary characteristics of six forests and eight ecoregions in the subtropical region of China

Forest types	*N*	*n*	D^2^H			AGB (kg)		
			Min	Max	Mean	Min	Max	Mean
*Cunninghamia lanceolata* forest (CL)	6	219	16.97	23,469.40	2878.59	1.25	358.95	49.86
Coniferous mixed broadleaf forest (CMBF)	6	77	20.39	9973.85	1587.48	1.35	156.32	42.84
Deciduous broadleaf forest (DBF)	5	40	50.69	9481.50	2157.55	2.76	240.26	59.28
Evergreen broadleaf forest (EBF)	6	277	80.69	42,360.52	4492.07	3.85	719.51	121.74
*Eucalyptus* tree species forest (ETS)	5	76	84.24	6149.86	1882.65	2.25	201.05	46.72
*Pinus massoniana* forest (PM)	7	283	16.25	19,822.5	2297.85	1.01	388.74	53.75

Ecoregions	*M*	*n*	MAT (°C)			MAP (mm)		
			Min	Max	Mean	Min	Max	Mean
Yangtze river delta ecological zone (ER1)	4	55	5.3	17.2	14.0	491	1381	907
Evergreen broadleaf forest ecological zone in the mountains of Zhejiang and Fujian provinces (ER2)	6	254	11.6	20.4	16.5	1022	1653	1342
Ecological zone in Jiangnan and Nanling mountains and hill (ER3)	6	185	13.0	20.7	16.9	1069	1626	1385
Evergreen broadleaf forest ecological zone in the mountains of the west Hunan, Guizhou and Hubei provinces (ER4)	6	161	7.4	17.7	14.8	798	1356	1073
Karst evergreen broadleaf forest and agricultural ecological zone in Guizhou and Guangxi provinces (ER5)	3	121	10.1	21.1	16.9	887	1493	1254
Ecological zone of Sichuan Basin (ER6)	2	39	7.3	18.1	15.4	677	1232	911
Ecological zone on Yunnan Plateau (ER7)	2	36	− 8.3	19.6	12.5	618	1129	831
South humid subtropical ecological zone (ER8)	6	121	6.9	22.9	19.1	833	1770	1252

Abbreviations in the brackets indicated each forest type and individual ecoregion, respectively in the subtropical region of China. Climate data, including mean annual temperature (MAT) and mean annual precipitation (MAP) was obtained from the National Climate Center (http://ncc.cma.gov.cn/cn/)

*N* number of forest distributed ecoregions, *n* number of sampling plots, *M* number of forest types

### The dataset

The AGB data of the six subtropical forests (CL, CMBF, DBF, EBF, ETS, and PM) was collected from the large forest biomass dataset in China, which was compiled from published biomass studies and pre-existing datasets published between 1978 and 2008 (Luo et al. 2013). Moreover, we reviewed almost all related publications in China from 2008 to 2013 and recorded information of sampling plots on locations, forest types, stand age, stand density, DBH, tree height, wood density and AGB for the six subtropical forest types. The biomass components of sample trees (stems, branches, leaves, etc.) was measured using destructive harvesting and oven weighing method. Then, AGB of average tree (kg) for each plot was calculated from stand AGB (Mg ha^−1^) and stand density. Our criteria for selected sampling tress was that the sample trees for each forest types were distributed as evenly as possible in the diameter classes in our dataset. Together, we collected 972 records of plot measured AGB data for the average trees of six subtropical forest types (Table 1; Additional file 1: Figure S1), 316 records of which had wood density information. The locations and forest types of the dataset were shown in Fig. 1.

### Model description

#### Allometric model at different scales

We used general allometric equations to estimate AGB of six subtropical forests at individual ecoregional scale (model 1) and all subtropical regional scale (model 3), respectively. These allometric equations based on *D* ^*2*^ *H* (*D*, diameter of the tree at breast height, cm, and *H*, tree height, m), which have been widely used to estimate AGB for forests (Jenkins et al. 2003; Muukkonen 2007; Návar 2009).

1 $${ \ln }\,(AGB) = a + b\ln\, (D^{2} H) + \varepsilon$$

where *AGB* is the aboveground biomass, *a* and *b* are parameters, and *ɛ* is the additive error. Then, the estimate of aboveground biomass is as follows:

2 $$AGB_{\text{est}} = \exp \left( {{{a + RSE^{2} } \mathord{\left/ {\vphantom {{a + RSE^{2} } 2}} \right. \kern-0pt} 2}} \right) \times \left( {D^{2} H} \right)^{b}$$

where *RSE* is the residual standard errors of the regressions.

We considered ecoregion as dummy variable and used dummy variable allometric model to estimate AGB for six subtropical forests at both ecoregion scale and regional scale (model 2). The general form of the dummy variable allometric model was as follows (Wang et al. 2008; Zeng et al. 2011).

3 $${ \ln }\,(AGB) = a_{0} + \sum {a_{i} z_{i} + } b\ln\, (D^{2} H) + \varepsilon$$

4 $$AGB_{\text{est}} = \exp \left( {{{a_{0} + \sum {a_{i} z_{i} } + RSE^{2} } \mathord{\left/ {\vphantom {{a_{0} + \sum {a_{i} z_{i} } + RSE^{2} } 2}} \right. \kern-0pt} 2}} \right) \times \left( {D^{2} H} \right)^{b}$$

where *z*_*i*_ is the dummy variable, *a*_*i*_ is the ecoregion-specific parameter, and other symbols are the same as Eqs. (1) and (2). The dummy variables are 0, 1. In model 2, we take each ecoregion as dummy variable, if forest has six distributed ecoregions, we used six dummy variables, z_1_, z_2_, z_3_, z_4_, z_5_ and z_6_, when z_1_ = 1, the others = 0, when z_2_ = 1, the others = 0, etc. (Zeng et al. 2011).

#### Testing the importance of wood density for accurate estimated AGB

At regional scale, we used 316 plot AGB data with wood density (*WD*) records to test whether *WD* improved accuracy of allometric model. *D* ^2^ *H* × *WD* as variable was used to fit allometric model compared with the model without *WD* variable (Chave et al. 2014; Xu et al. 2015).

5 $${ \ln }\,(AGB) = a + b\ln \,(D^{2} H \times WD) + \varepsilon$$

then, the estimate of biomass is as follows:

6 $$AGB_{\text{est}} = \exp \left( {{{a + RSE^{2} } \mathord{\left/ {\vphantom {{a + RSE^{2} } 2}} \right. \kern-0pt} 2}} \right) \times \left( {D^{2} H \times WD} \right)^{b}$$

#### Accuracy assessment of models

The coefficient of determination (*R* ^2^), residual standard error of the regression (*RSE*), and mean percent standard error (*MPSE*) were used to assess the accuracies of the models (Zeng et al. 2011). *MPSE* were defined as follows:

7 $$MPSE = \frac{1}{n}\sum {\left| {{{({\text{y}}_{i} - \hat{y})} \mathord{\left/ {\vphantom {{({\text{y}}_{i} - \hat{y})} {\hat{y}}}} \right. \kern-0pt} {\hat{y}}}} \right|} \times 1 0 0$$

where *n* is number of plots; *y*_*i*_ and $\hat{y}$ are the observed and estimated values of AGB respectively.

## Results

### Allometric models for aboveground biomass estimation at different scales

The values of AGB and D^2^H varied obviously in each forest type and each ecoregion, especially in DBF, ETS, CMBF and EBF (Table 1; Additional file 1: Figure S1). There was clear distinction of climate (e.g., MAT and MAP) among eight ecoregions (Table 1).

Model 1 had better performance for CL in ER4 (*R* ^2^ = 0.987) and ER5 (*R* ^2^ = 0.986), for CMBF in ER4 (*R* ^2^ = 0.978) and ER6 (*R* ^2^ = 0.961), for DBF in ER1 (*R* ^2^ = 0.975) and ER4 (*R* ^2^ = 0.961), for EBF in ER7 (*R* ^2^ = 0.978) and ER4 (*R* ^2^ = 0.968), for ETS in ER2 (*R* ^2^ = 0.999) and ER4 (*R* ^2^ = 0.979), and for PM in ER5 (*R* ^2^ = 0.986), ER6 (*R* ^2^ = 0.979), ER3 (*R* ^2^ = 0.970) and ER2 (*R* ^2^ = 0.960), and showed lower performance for CMBF in ER2 (*R* ^2^ = 0.889), and for EBF in ER8 (*R* ^2^ = 0.885) (Table 2; Additional file 1: Figure S2).

**Table 2:** Parameters of allometric models for estimating aboveground biomass of six forests at individual ecoregion scale in the subtropical region of China

Forests	Ecoregions	Allometric models at individual ecoregion scale (model 1)	*n*	*R* ^2^	*RSE*	*F* value
CL	ER1	exp(− 3.006 + 0.008) × (*D* ^2^ *H*)^0.857^	14	0.918	0.123	134.9***
	ER2	exp(− 2.065 + 0.029) × (D^2^H)^0.762^	92	0.946	0.241	1574.6***
	ER3	exp(− 1.431 + 0.034) × (D^2^H)^0.669^	57	0.925	0.260	679.1***
	ER4	exp(− 1.659 + 0.009) × (D^2^H)^0.688^	18	0.987	0.138	1203.8***
	ER5	exp(− 3.611 + 0.017) × (D^2^H)^0.936^	12	0.986	0.184	690.6***
	ER8	exp(− 2.067 + 0.033) × (D^2^H)^0.749^	26	0.954	0.256	499.8***
CMBF	ER1	exp(− 4.280 + 0.011) × (D^2^H)^1.169^	7	0.955	0.148	104.8***
	ER2	exp(− 1.746 + 0.057) × (D^2^H)^0.744^	34	0.889	0.338	255.6***
	ER3	exp(− 0.527 +0.044) × (D^2^H)^0.552^	7	0.912	0.295	51.5***
	ER4	exp(− 0.615 + 0.001) × (D^2^H)^0.590^	7	0.978	0.053	222.3***
	ER6	exp(− 1.953 + 0.043) × (D^2^H)^0.777^	14	0.961	0.294	293.6***
	ER8	exp(− 1.391 + 0.048) × (D^2^H)^0.72^	8	0.932	0.102	82.2***
DBF	ER1	exp(− 2.054 + 0.019) × (D^2^H)^0.803^	11	0.975	0.195	305.3***
	ER2	exp(− 2.202 + 0.036) × (D^2^H)^0.774^	11	0.955	0.270	191.6***
	ER3	exp(− 14.935 + 0.106) × (D^2^H)^2.623^	5	0.904	0.460	28.2*
	ER4	exp(− 4.803 + 0.020) × (D^2^H)^1.120^	7	0.961	0.198	122.9***
	ER8	exp(− 0.841 + 0.034) × (D^2^H)^0.722^	6	0.908	0.262	39.3**
EBF	ER2	exp(− 2.909 + 0.054) × (D^2^H)^0.920^	55	0.932	0.328	729.7***
	ER3	exp(− 0.482 + 0.022) × (D^2^H)^0.592^	29	0.901	0.209	245.2***
	ER4	exp(− 1.699 + 0.011) × (D^2^H)^0.820^	48	0.968	0.149	1372.4***
	ER5	exp(− 0.610 + 0.023) × (D^2^H)^0.656^	89	0.913	0.216	909.0***
	ER7	exp(− 2.067 + 0.004) × (D^2^H)^0.817^	28	0.978	0.086	1168.7***
	ER8	exp(− 2.761 + 0.068) × (D^2^H)^0.911^	28	0.885	0.368	200.8***
ETS	ER2	exp(− 1.305 + 0.000) × (D^2^H)^0.687^	9	0.999	0.017	5074.8***
	ER3	exp(− 5.646 + 0.029) × (D^2^H)^1.245^	12	0.900	0.240	90.0***
	ER4	exp(− 3.615 + 0.001) × (D^2^H)^0.859^	6	0.979	0.044	184.0***
	ER7	exp(− 1.352 + 0.021) × (D^2^H)^0.631^	8	0.924	0.204	73.3***
	ER8	exp(− 3.062 + 0.026) × (D^2^H)^0.905^	41	0.904	0.230	368.8***
PM	ER1	exp(− 2.515 + 0.020) × (D^2^H)^0.843^	23	0.948	0.201	379.7***
	ER2	exp(− 2.071 + 0.040) × (D^2^H)^0.804^	53	0.960	0.283	1214.7***
	ER3	exp(− 2.176 + 0.036) × (D^2^H)^0.798^	75	0.970	0.217	2374.4***
	ER4	exp(− 2.589 + 0.037) × (D^2^H)^0.839^	75	0.942	0.271	1181.8***
	ER5	exp(− 3.448 + 0.011) × (D^2^H)^0.973^	20	0.986	0.146	1301.2***
	ER6	exp(− 2.366 + 0.018) × (D^2^H)^0.831^	25	0.979	0.187	1058.0***
	ER8	exp(− 2.030 + 0.081) × (D^2^H)^0.797^	12	0.935	0.403	143.1***

*** Indicates significant at *p* < 0.001 level; ** indicates significant at *p* < 0.01 level, * indicates significant at *p* < 0.05 level. See Table 1 for the abbreviations of the forest types and ecoregions

Model 2 considered the effects of the ecoregions on AGB estimation. The statistical results showed ER2 significantly influenced AGB estimations for CL (*p* < 0.05), and for PM (*p* < 0.01), respectively. ER8 significantly affected on AGB estimations for DBF (*p* < 0.001), EBF (*p* < 0.001), CMBF (*p* < 0.05) and ETS (*p* < 0.05). ER4, ER5, ER3, and ER8 significantly affected on AGB estimation for EBF, especially ER4 and ER5 (*p* < 0.0001) (Table 3; Additional file 1: Figure S3).

**Table 3:** Parameters of dummy variable allometric model for estimating aboveground biomass of six forest types at both regional scale and ecoregion scale in the subtropical region of China

Forests	Ecoregions	Dummy variable allometric model (model 2)	*R* ^2^	*RSE*	*F* value
CL	General	exp(− 2.064 + 0.166 + 0.142 + 0.083 − 0.009 + 0.071 + 0.031) × (D^2^H)^0.739^	0.947	0.249	631.5***
	ER1	exp(− 2.064 + 0.031) × (D^2^H)^0.739^			
	ER2*	exp(− 2.064 + 0.166 + 0.031) × (D^2^H)^0.739^			
	ER3	exp(− 2.064 + 0.142 + 0.031) × (D^2^H)^0.739^			
	ER4	exp(− 2.064 + 0.083 + 0.031) × (D^2^H)^0.739^			
	ER5	exp(− 2.064 − 0.009 + 0.031) × (D^2^H)^0.739^			
	ER8	exp(− 2.064 + 0.071 + 0.031) × (D^2^H)^0.739^			
CMBF	General	exp(− 1.391 − 0.18 − 0.143 −0.134 − 0.239 + 0.398 + 0.048) × (D^2^H)^0.720^	0.920	0.311	134.5***
	ER1	exp(− 1.391 + 0.048) × (D^2^H)^0.720^			
	ER2	exp(− 1.391 − 0.180 + 0.048) × (D^2^H)^0.720^			
	ER3	exp(− 1.391 − 0.143 + 0.048) × (D^2^H)^0.720^			
	ER4	exp(− 1.391 − 0.134 + 0.048) × (D^2^H)^0.720^			
	ER6	exp(− 1.391 − 0.239 + 0.048) × (D^2^H)^0.720^			
	ER8*	exp(− 1.391 + 0.398 + 0.048) × (D^2^H)^0.720^			
DBF	General	exp(− 2.191 − 0.338 + 0.328 − 0.177 + 0.803 + 0.045) × (D^2^H)^0.823^	0.940	0.301	106.6***
	ER1*	exp(− 2.191 + 0.045) × (D^2^H)^0.823^			
	ER2	exp(− 2.191 − 0.338 + 0.045) × (D^2^H)^0.823^			
	ER3	exp(− 2.191 + 0.328 + 0.045) × (D^2^H)^0.823^			
	ER4	exp(− 2.191 − 0.177 + 0.045) × (D^2^H)^0.823^			
	ER8***	exp(− 2.191 + 0.803 + 0.045) × (D^2^H)^0.823^			
EBF	General	exp(− 1.896 − 0.141 + 0.451 + 0.321 + 0.103 + 0.184 + 0.038) × (D^2^H)^0.785^	0.915	0.277	481.8***
	ER2	exp(− 1.896 + 0.038) × (D^2^H)^0.785^			
	ER3*	exp(− 1.896 −0.141 + 0.038) × (D^2^H)^0.785^			
	ER4***	exp(− 1.896 + 0.451 + 0.038) × (D^2^H)^0.785^			
	ER5***	exp(− 1.896 + 0.321 + 0.038) × (D^2^H)^0.785^			
	ER7	exp(− 1.896 + 0.103 + 0.038) × (D^2^H)^0.785^			
	ER8**	exp(− 1.896 + 0.184 + 0.038) × (D^2^H)^0.785^			
ETS	General	exp(− 2.495 − 0.015 − 1.918 − 0.221 − 0.232 + 0.028) × (D^2^H)^0.859^	0.954	0.237	288.5***
	ER2	exp(− 2.495 + 0.028) × (D^2^H)^0.859^			
	ER3	exp(− 2.495 − 0.015 +0.028) × (D^2^H)^0.859^			
	ER4***	exp(− 2.495 − 1.918 + 0.028) × (D^2^H)^0.859^			
	ER7*	exp(− 2.248 − 0.221 + 0.028) × (D^2^H)^0.859^			
	ER8*	exp(− 2.248 − 0.232 + 0.028) × (D^2^H)^0.859^			
PM	General	exp(− 2.369 + 0.168 + 0.053 − 0.102 + 0.048 + 0.072 + 0.156 + 0.032) × (D^2^H)^0.821^	0.964	0.252	1055.0***
	ER1	exp(− 2.369 + 0.032) × (D^2^H)^0.821^			
	ER2**	exp(− 2.369 + 0.168 + 0.032) × (D^2^H)^0.821^			
	ER3	exp(− 2.369 + 0.053 + 0.032) × (D^2^H)^0.821^			
	ER4	exp(− 2.369 − 0.102 + 0.032) × (D^2^H)^0.821^			
	ER5	exp(− 2.369 + 0.048 + 0.032) × (D^2^H)^0.821^			
	ER6	exp(− 2.369 + 0.072 + 0.032) × (D^2^H)^0.821^			
	ER8	exp(− 2.369 + 0.156 + 0.032) × (D^2^H)^0.821^			

*** Indicates significant at *p* < 0.001 level; ** indicates significant at *p* < 0.01 level; * indicates significant at *p* < 0.05 level. See Table 1 for the abbreviations of the forest types and ecoregions

Model 2 had better performance to estimate AGB for PM (*R* ^2^ = 0.964), ETS (*R* ^2^ = 0.954), and CL (*R* ^2^ = 0.947), and showed lower performance to estimate AGB for EBF (R^2^ = 0.915) and CMBF (*R* ^2^ = 0.920) (Table 3; Additional file 1: Figure S3).

Model 3 showed better performance to estimate AGB for PM (*R* ^2^ = 0.959) and CL (*R* ^2^ = 0.944), and had lower performance to estimate AGB for the other four forest types, especially for ETS (*R* ^2^ = 0.759) (Table 4; Additional file 1: Figure S4).

**Table 4:** Parameters of allometric models for estimating aboveground biomass of six forests at regional ecoregion scale in the subtropical region of China

Forests	Allometric model at regional scale (model 3)	*n*	*R* ^2^	*RSE*	*F* value
CL	exp(− 1.920 + 0.032) × (D^2^H)^0.736^	219	0.944	0.252	3668.5***
CMBF	exp(− 1.485 + 0.062) × (D^2^H)^0.718^	77	0.890	0.353	607.4***
DBF	exp(− 1.536 + 0.110) × (D^2^H)^0.733^	40	0.839	0.466	198.3***
EBF	exp(− 1.481 + 0.056) × (D^2^H)^0.757^	277	0.874	0.334	1905.5***
ETS	exp(− 3.776 + 0.138) × (D^2^H)^0.995^	76	0.759	0.526	233.4***
PM	exp(− 2.394 + 0.036) × (D^2^H)^0.830^	283	0.959	0.267	6547.2***

*** Indicates significant at *p* < 0.001 level. See Table 1 for the abbreviations of the forest types

### Assessment the accuracies of the allometric models at different scales

**Fig. 2:**
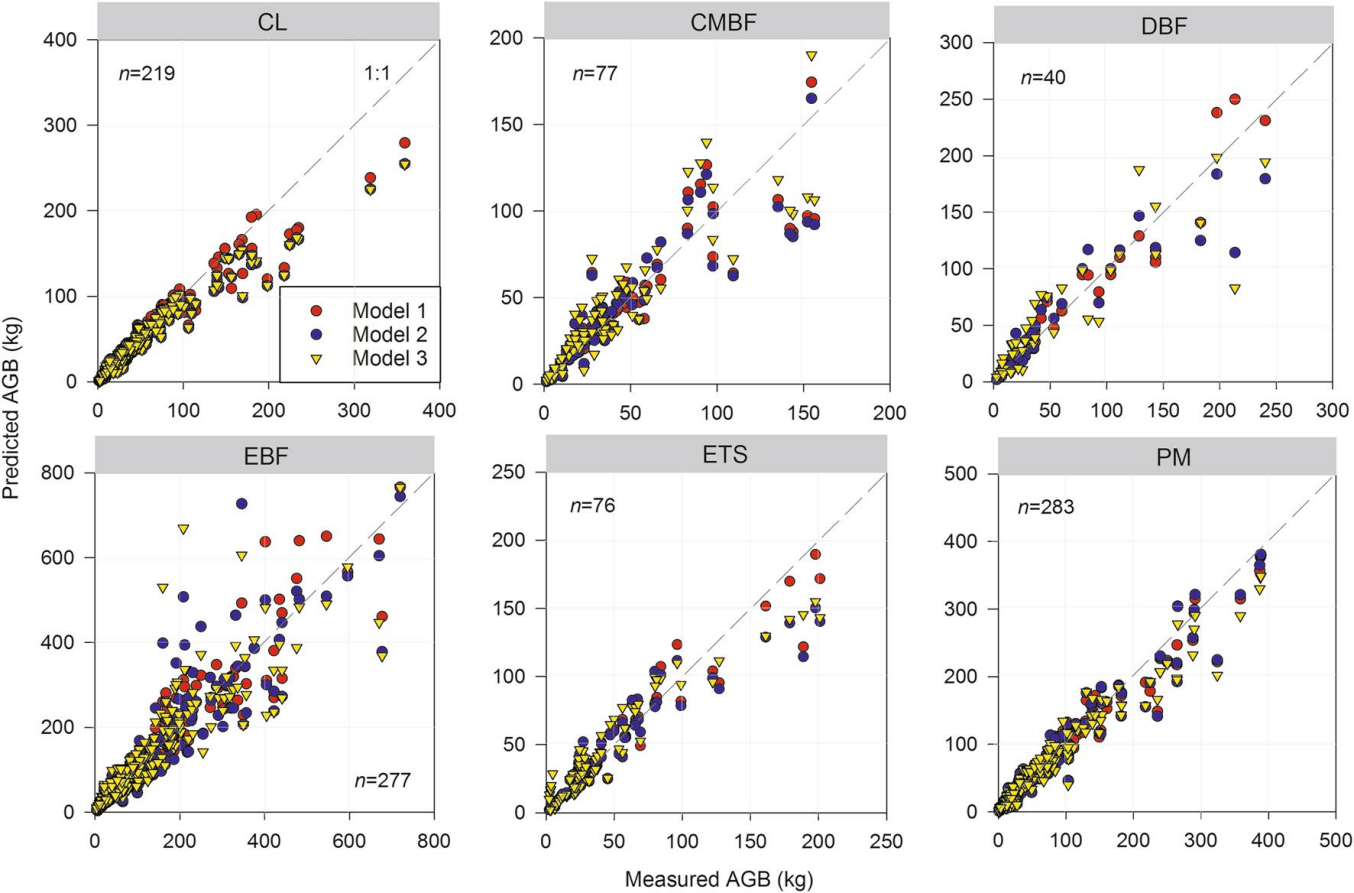
Comparison measured AGB (aboveground tree biomass) and predicted AGB for six subtropical forests from three allometric models at different scales (model 1, model 2 and model 3) in China. Model 1: allometric model at ecoregion scale, model 2: dummy variable allometric model at both ecoregion scale and regional scale, and model 3: allometric model at regional scale. See Table 1 for the abbreviations of the six forest types

We compared measured AGB and predicted AGB estimated from three allometric models at different scales, and found that model 1 and model 2 had better accuracies for AGB estimations than model 3 (Fig. 2, Table 5). The *MPSE* values of three models varied obviously for AGB estimations in the same forest in different ecoregions, and showed increasing trend with increasing scales. At ecoregional scale, the values of *MPSE* from model 2 were similar to model 1, less than model 3 for CL, PM and DBF in the distributed ecoregions except for CL and PM in ER5, and for DBF in ER3 and ER4. For EBF and ETS, *MPSE* showed the same trend in the ecoregions except for EBF in ER1 and ETS in ER2. For all the forests, *MPSE* of Model 1, model 2, and model 3 ranged from 2.79 to 30.40%, 5.15 to 40.94%, and 13.25 to 80.81% at ecoregional scale, respectively. At regional scale, *MPSE* of model 2 was very similar to model 1, and was clearly less than model 3 in six subtropical forests (Table 5).

**Table 5:** *MPSE* for three allometric models at different scales developed in the subtropical region of China

Forests	Models	*MPSE* (%)								
		ER1	ER2	ER3	ER4	ER5	ER6	ER7	ER8	Region
CL	Model 1	9.51	19.38	20.26	10.06	11.72			20.41	17.92
	Model 2	10.83	19.27	21.49	11.71	29.08			21.07	19.44
	Model 3	26.04	21.66	23.99	21.99	27.36			24.02	23.01
CMBF	Model 1	9.66	26.40	17.89	3.64		22.08		7.75	19.31
	Model 2	20.97	26.16	30.69	7.45		25.55		23.19	23.98
	Model 3	20.68	28.28	28.79	13.25		31.29		53.38	29.42
DBF	Model 1	12.95	21.70	14.00	12.89				14.61	15.73
	Model 2	12.53	23.90	40.94	25.35				17.67	22.22
	Model 3	18.46	37.84	60.54	29.13				72.78	39.07
EBF	Model 1		25.24	13.26	9.82	16.41		5.61	30.40	17.02
	Model 2		30.74	17.25	9.87	18.32		5.29	30.65	19.14
	Model 3		32.27	29.85	24.61	21.70		11.01	31.03	25.02
ETS	Model 1		0.97	18.77	2.79			16.48	17.65	14.38
	Model 2		8.20	26.37	5.15			20.46	17.54	16.96
	Model 3		29.45	23.77	80.81			39.21	20.15	28.28
PM	Model 1	14.45	18.94	15.85	20.86	11.92	15.32		27.19	17.80
	Model 2	14.63	19.73	16.05	20.75	19.16	15.39		28.35	18.55
	Model 3	15.18	24.97	16.59	23.05	18.40	15.38		33.35	20.49

See Table 1 for the abbreviations of six forests and eight ecoregions

### Testing importance of wood density for Aboveground biomass estimation

**Fig. 3:**
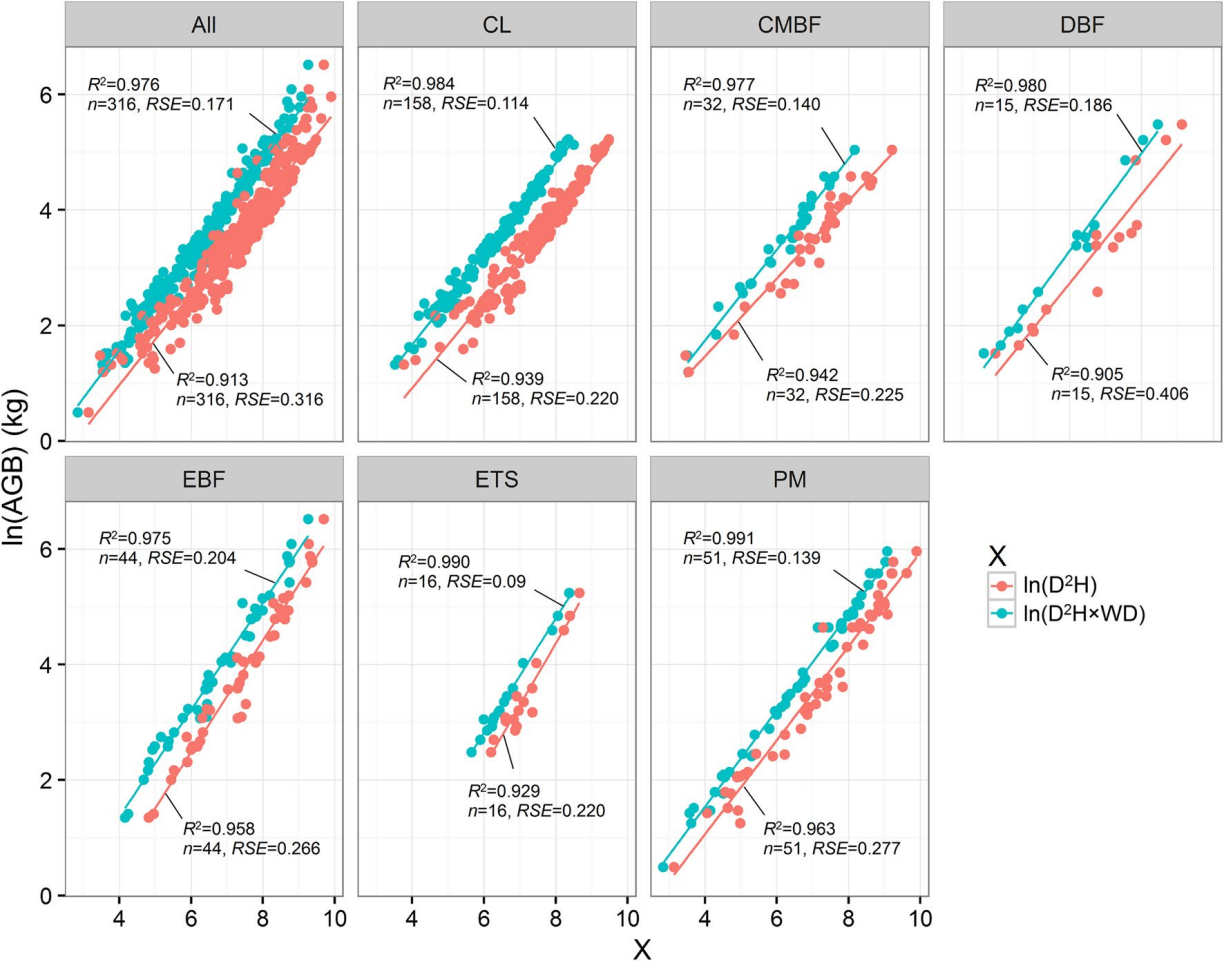
Fitted curves for each forest type and all forest types at regional scale in subtropical region of China applied the allometric model with wood density variable, and the allometric model without wood density variable. See Table 1 for the abbreviations of the six forest types

**Fig. 4:**
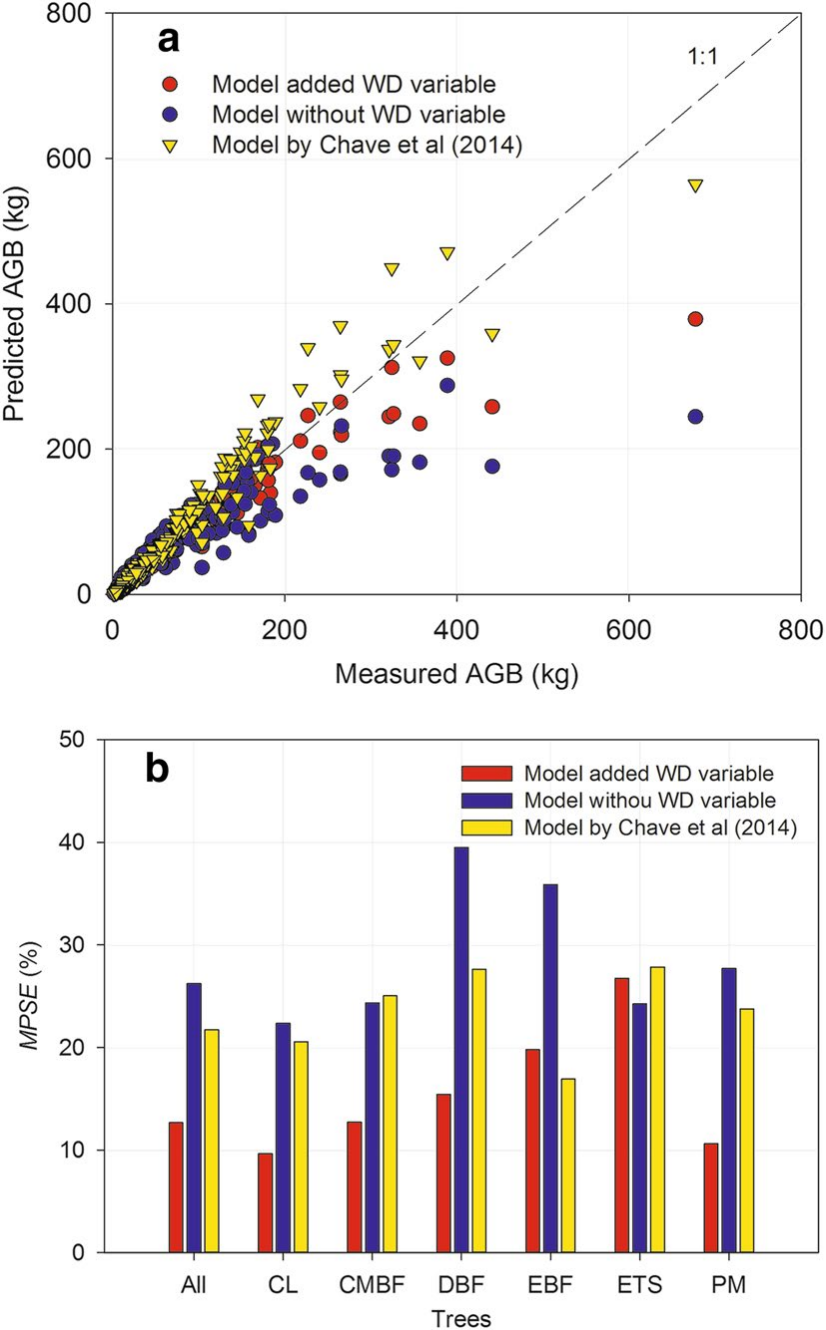
Compared the measured AGB (aboveground tree biomass) and the estimated AGB in subtropical forests by the allometric model with wood density, the allometric model without wood density, and the model widely used in tropical trees created by Chave et al. (2014) $\left( {{\text{AGB}}_{\text{est}} \; = \;0.0 6 7 3\; \times \; \left( {\rho D^{ 2} H} \right)^{0. 9 7 6} } \right)$, respectively (**a**), and compared *MPSE* among these three allometric models (**b**)

Figure 3 showed that WD variable used in allometric model greatly improved the estimate accuracies with higher *R* ^2^, lower *RSE* and *MPSE* than traditional allometric model without WD variable for six forests (Figs. 3, 4). The allometric model with WD variable developed by Chave et al. (2014) showed lower *MPSE* than traditional model for CL, DBF, EBF and PM, especially in EBF and DBF, and showed greater *MPSE* for AGB estimations in CL, CMBF, DBF, ETS and PM than allometric model with WD variable from our dataset (Fig. 4). In EBF, model created by Chave et al. (2014) showed similar lower *MPSE* to our model with WD variable (Fig. 4).

## Discussion

### Allometric models for aboveground biomass estimation at different scales

Many scientists gave efforts to improve the tree allometric models at single tree, plot, regional, national, or even worldwide scales (Brown et al. 1989; Chave et al. 2005; Návar 2009; Genet et al. 2011). In this study, we developed three allometric models from ecoregion to regional scales. Three allometric models using *D* ^2^ *H* as the predictive variable offered good fitness of AGB allometric models at different scales (*R* ^2^ ranged from 0.759 to 0.999) (Tables 2, 3, 4). This indicates *D* ^2^ *H* as variable could improve accuracy of models (Muukkonen 2007; Návar 2009; Xu et al. 2015). Muukkonen (2007) found that allometric equations with only DBH as an independent variable provided lower overall estimations of tree biomass. Models at different scales may lead to variation of biomass estimation because of difference of climatic conditions, site quality, and forest structures (Muukkonen 2007; Fu et al. 2017). In this study, we found that model 1 and model 2 had better accuracies for AGB estimations than model 3 (Fig. 2; Table 3). The *MPSE* values of three models varied obviously for AGB estimations in the same forest in different ecoregions, and showed increasing trend with increasing scales. Case and Hall (2008) found that prediction error of generalized tree biomass equations for ten species in the boreal forest region of west-central Canada increased from regional to national scale. However, *MPSE* of model 2 was very similar to model 1, and obviously less than model 3 in six forests (Table 5), which indicated that dummy variable allometric model considered ecoregion factors could be proposed as general model to estimate AGB for subtropical forests, and provide a more effective new approach to improve the compatibility of forest biomass estimates at the ecoregional, and regional scales.

### Assessment the accuracies of the allometric models at different scales

Regional climate data affected the precision of the regional model (Drake et al. 2003; Dewalt and Chave 2004; Chave et al. 2005; Wang 2006; Fu et al. 2017). In this study, MAT and MAP were clearly distinct among eight ecoregions (Table 1). Ecoregions including ER2, ER3, ER4, ER5, and ER8 significantly affected AGB estimations (Table 3). The aboveground biomass of PM, CL, and ETS was greater in the southern central regions with higher temperature and greater rainfall, than in the west regions with lower temperatures and less rainfall. The influences of climate were even more significant in EBF (Table 3). Therefore, forest regional climate data should be considered when the regional models were employed (Muukkonen 2007; Fu et al. 2017).

The number of plots applied to develop the allometric equations in ETS, DBF, and CBMF forest types (*N* < 100) may not be enough to represent the full range of species present at the study areas (Table 1). Návar (2009) reported that several hundred sampling plots were needed for fitting regional allometric equations. Moreover, three model at different scales that have been developed were more robust when there were not enough trees with diameters between 25 and 40 cm (Additional file 1: Figure S1), and the majority of samples had insufficient trees with a diameter of more than 25 cm, which would lead larger estimated error of AGB (Wang 2006; Zaehle et al. 2006; Hudiburg et al. 2009; Xiang et al. 2016).

### Testing importance of wood density for aboveground biomass estimation

Wood density strongly varies among different geographical regions, climate gradients, and correlated to forest structure, tree architecture (Baker et al. 2004; Chave et al. 2005, 2014). Thus, wood density can improve the performance of allometric model. In this study, we compared the performance of the allometric model with wood density variable with traditional model without wood density variable at regional scale, and the model created by Chave et al. (2014), and found the model with wood density variable had better performance than other two models (Figs. 3, 4). It indicated that taking wood density as variables in the allometric model could greatly improve accuracy of biomass model (Chave et al. 2014; Xu et al. 2015). The reason was that wood density could reflect site climate, forest structure, and trees architecture, and reduce the effects of site climate and forest structure on AGB estimations. The model created by Chave et al. (2014) showed better performance for AGB estimation of EBF, similar to our model with wood density variable. This result suggests that the model created by Chave et al. (2014) can be used AGB estimation of EBF in subtropical region of China.

## Conclusions

This study showed that ecoregions significantly affected AGB estimation for six subtropical forests in China. Dummy variable allometric model considered ecoregion as dummy variable had better performance similar to allometric model at both individual ecoregional scale and regional scale. Furthermore, we tested the performance of allometric model with wood density at regional scale and found wood density as an important variable in the allometric models greatly improved the accuracies of AGB estimations in six subtropical forests. Our findings showed that dummy variable allometric model considered ecoregion factors could be proposed as general model to estimate AGB for subtropical forests, and provide a more effective new approach to improve the compatibility of forest biomass estimates at the ecoregional, and regional scales. Moreover, the new allometric models with wood density, diameter, and tree height were more accurate than the traditional models without wood density in AGB estimations for subtropical forests at regional scales.

## Additional file

**Additional file 1: Figure S1.** Observed AGB (aboveground biomass) plotted with D^2^H (D, diameter at tree breast height, H, tree height) of six forests distributed in eight ecoregions of subtropical region in China. **Figure S2.** Fitted curves for six forests applied allometric model at each ecoregion scale (model 1) in the subtropical region of China. **Figure S3.** Fitted curves for six forests applied dummy variable allometric model at both regional scale and ecoregion scale (model 2) in the subtropical region of China. **Figure S4.** Fitted curves for six subtropical forests applied allometric model at regional scale (model 3) in China.
