# Supplementary material for: Aboveground biomass estimation at different scales for subtropical forests in China
Source: Bot Stud. 2017 Nov 9;58:45. doi: 10.1186/s40529-017-0199-1 (PMC5680411; doi:10.1186/s40529-017-0199-1)
Supplement: Supplementary file 1 — Additional file 1: Figure S1. Observed AGB (aboveground biomass) plotted with D2H (D, diameter at tree breast height, H, tree height) of six forests distributed in eight ecoregions of subtropical region in China. Figure S2. Fitted curves for six forests applied allometric model at each ecoregion scale (model 1) in the subtropical region of China. Figure S3. Fitted curves for six forests applied dummy variable allometric model at both regional scale and ecoregion scale (model 2) in the subtropical region of China. Figure S4. Fitted curves for six subtropical forests applied allometric model at regional scale (model 3) in China. [file 40529_2017_199_MOESM1_ESM.doc]

**Aboveground biomass estimation at different scales for subtropical forests in China**

Shunlei Peng ^1,2^, Nianpeng He ^1^,^*^ Guirui Yu ^1^, Qiufeng Wang ^1^

1 Key Laboratory of Ecosystem Network Observation and Modeling, Institute of Geographic Sciences and Natural Resources Research, CAS, Beijing 100101, PR China

2 Key laboratory of Ecological Restoration in the Hilly Area, Pingdingshan University, Pingdingshan, He’nan 467000.

Short title: Aboveground biomass estimation at different scales

* Corresponding author. G.R. Yu

Institute of Geographic Sciences and Natural Resources Research, CAS

Beijing 100101

PR China

Tel. 010-64889263

E-mail: [yugr@igsnrr.ac.cn](mailto:yugr@igsnrr.ac.cn)

**Additional file**

**Figure S1** Observed AGB (aboveground biomass) plotted with D^2^H (D, diameter at tree breast height, H, tree height) of six forests distributed in eight ecoregions of subtropical region in China.


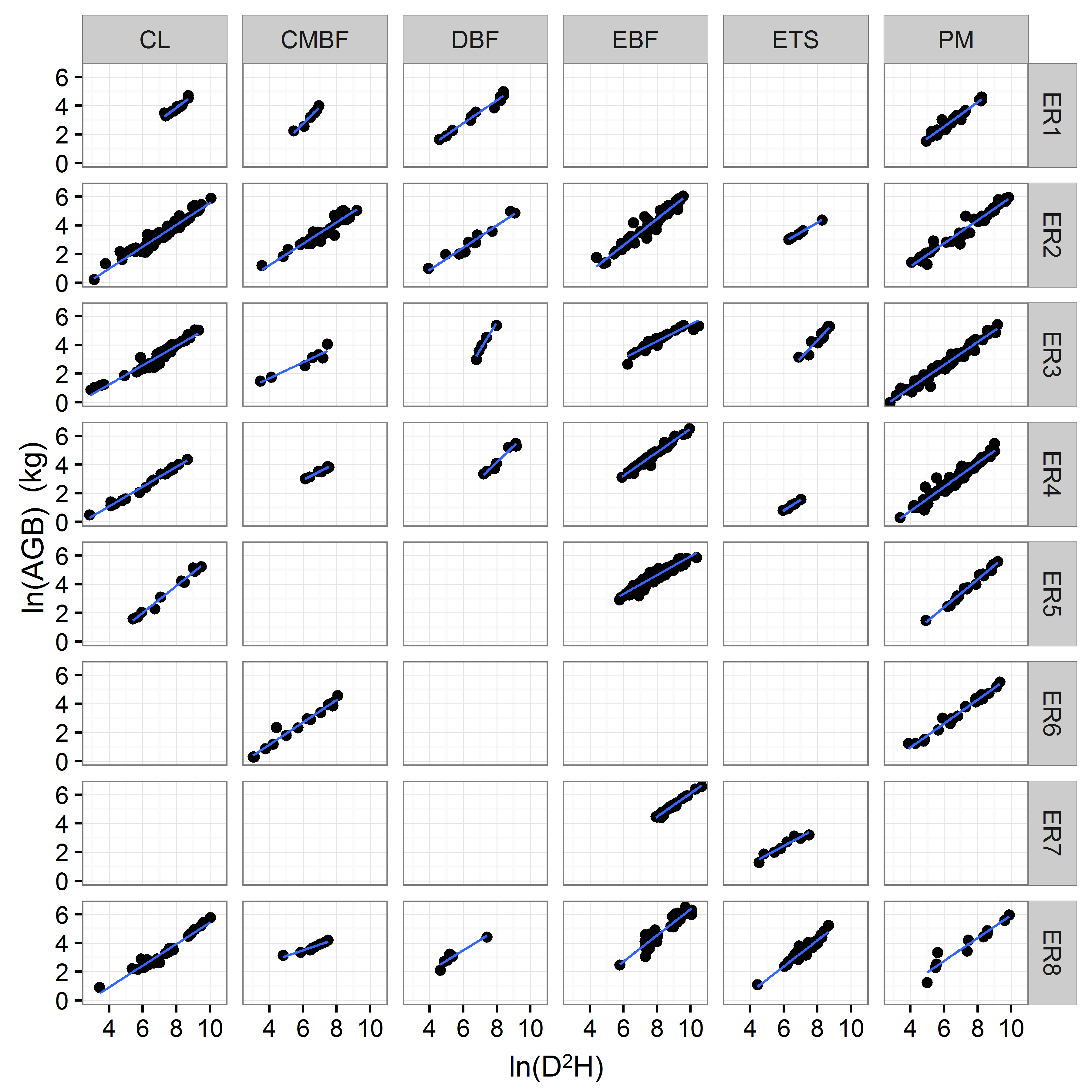


ER1: Yangtze River delta ecological zone, ER2: Evergreen broad leaved forest ecological zone in the mountains of Zhejiang and Fujian provinces, ER3: Ecological zone in Jiangnan and Nanling mountains and hills, ER4: Evergreen broadleaf forest ecological zone in the mountains of the west Hunan, Guizhou and Hubei provinces, ER5: Karst evergreen broadleaf forest and agricultural ecological zone in Guizhou and Guangxi provinces, ER6: Ecological zone of Sichuan Basin, ER7: Ecological zone on Yunnan Plateau, ER8: South humid subtropical ecological zone.

CL: *Cunninghamia lanceolata* forest, CMBF: Coniferous and broadleaf mixed forest, DBF: Subtropical deciduous broadleaf forest, EBF: Evergreen broadleaf forest, ETS: *Eucalyptus* tree species forest, and PM: *Pinus massoniana* forest.

**Figure S2** Fitted curves for six forests applied allometric model at each ecoregion scale (model 1) in the subtropical region of China


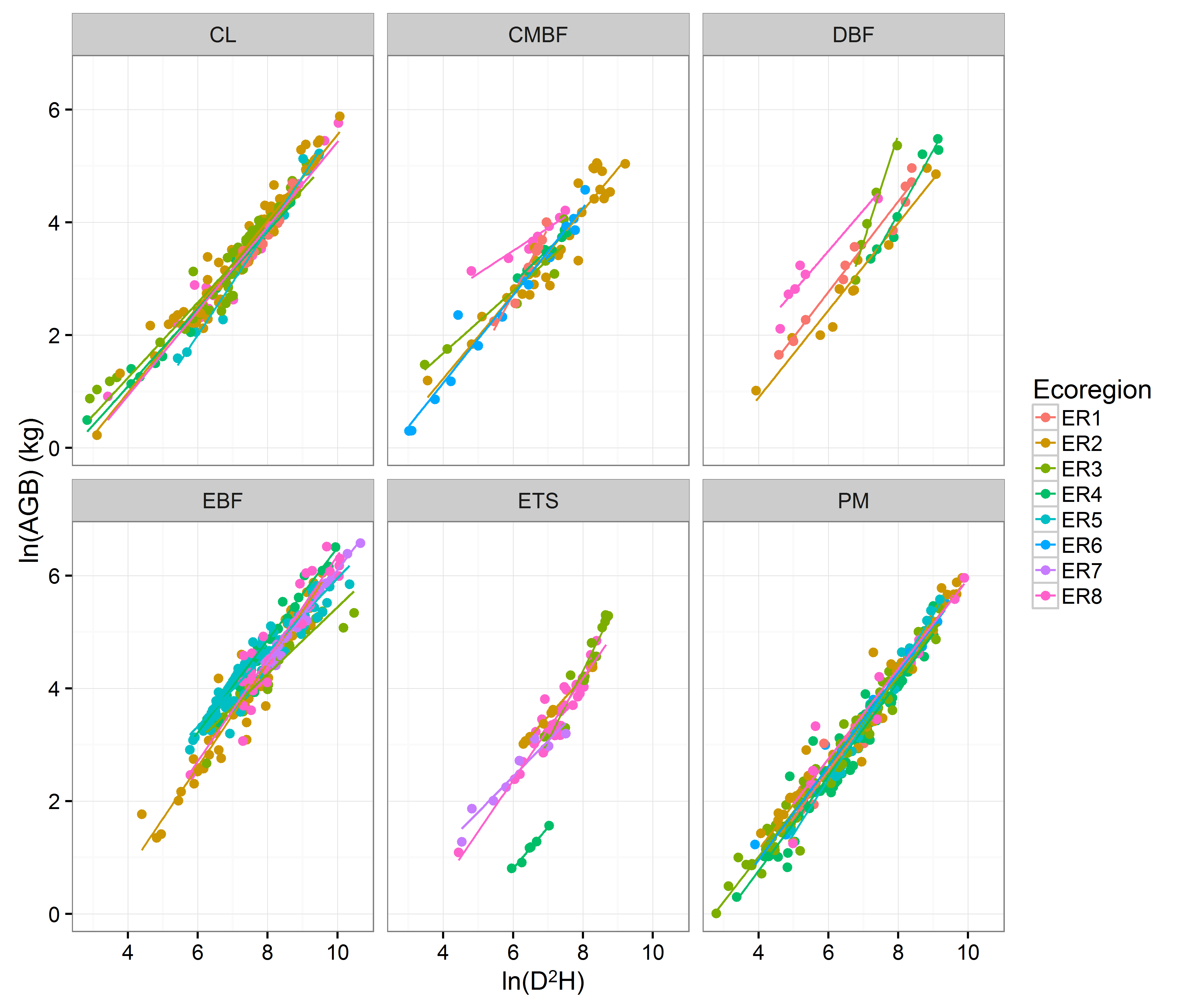


ER1: Yangtze River delta ecological zone, ER2: Evergreen broad leaved forest ecological zone in the mountains of Zhejiang and Fujian provinces, ER3: Ecological zone in Jiangnan and Nanling mountains and hills, ER4: Evergreen broadleaf forest ecological zone in the mountains of the west Hunan, Guizhou and Hubei provinces, ER5: Karst evergreen broadleaf forest and agricultural ecological zone in Guizhou and Guangxi provinces, ER6: Ecological zone of Sichuan Basin, ER7: Ecological zone on Yunnan Plateau, ER8: South humid subtropical ecological zone.

CL: *Cunninghamia lanceolata* forest, CMBF: Coniferous and broadleaf mixed forest, DBF: Subtropical deciduous broadleaf forest, EBF: Evergreen broadleaf forest, ETS: *Eucalyptus* tree species forest, and PM: *Pinus massoniana* forest.

**Figure S3** Fitted curves for six forests applied dummy variable allometric model at both regional scale and ecoregion scale (model 2) in the subtropical region of China.


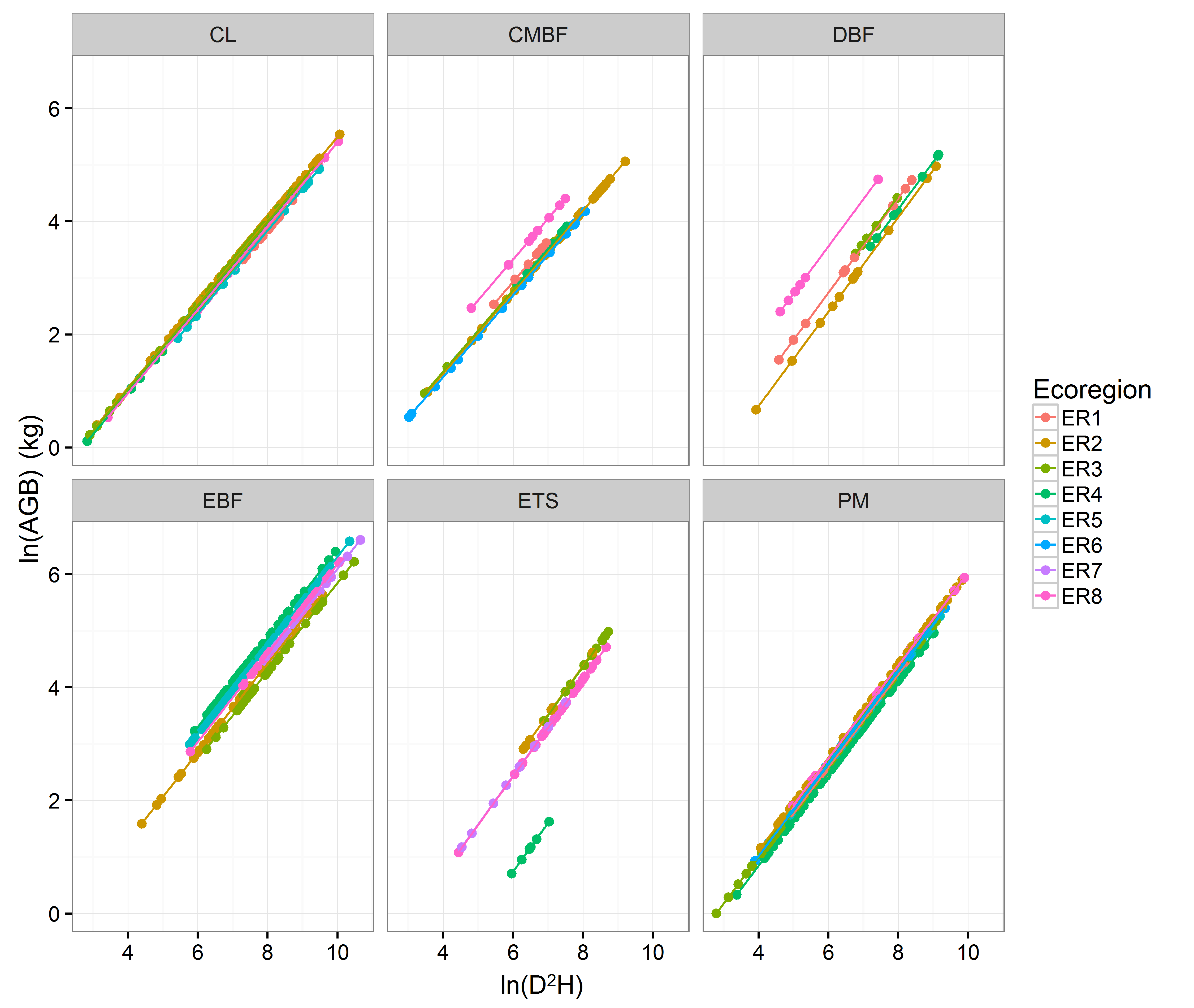


ER1: Yangtze River delta ecological zone, ER2: Evergreen broad leaved forest ecological zone in the mountains of Zhejiang and Fujian provinces, ER3: Ecological zone in Jiangnan and Nanling mountains and hills, ER4: Evergreen broadleaf forest ecological zone in the mountains of the west Hunan, Guizhou and Hubei provinces, ER5: Karst evergreen broadleaf forest and agricultural ecological zone in Guizhou and Guangxi provinces, ER6: Ecological zone of Sichuan Basin, ER7: Ecological zone on Yunnan Plateau, ER8: South humid subtropical ecological zone.

CL: *Cunninghamia lanceolata* forest, CMBF: Coniferous and broadleaf mixed forest, DBF: Subtropical deciduous broadleaf forest, EBF: Evergreen broadleaf forest, ETS: *Eucalyptus* tree species forest, and PM: *Pinus massoniana* forest.

**Figure S4** Fitted curves for six subtropical forests applied allometric model at regional scale (model 3) in China.

**
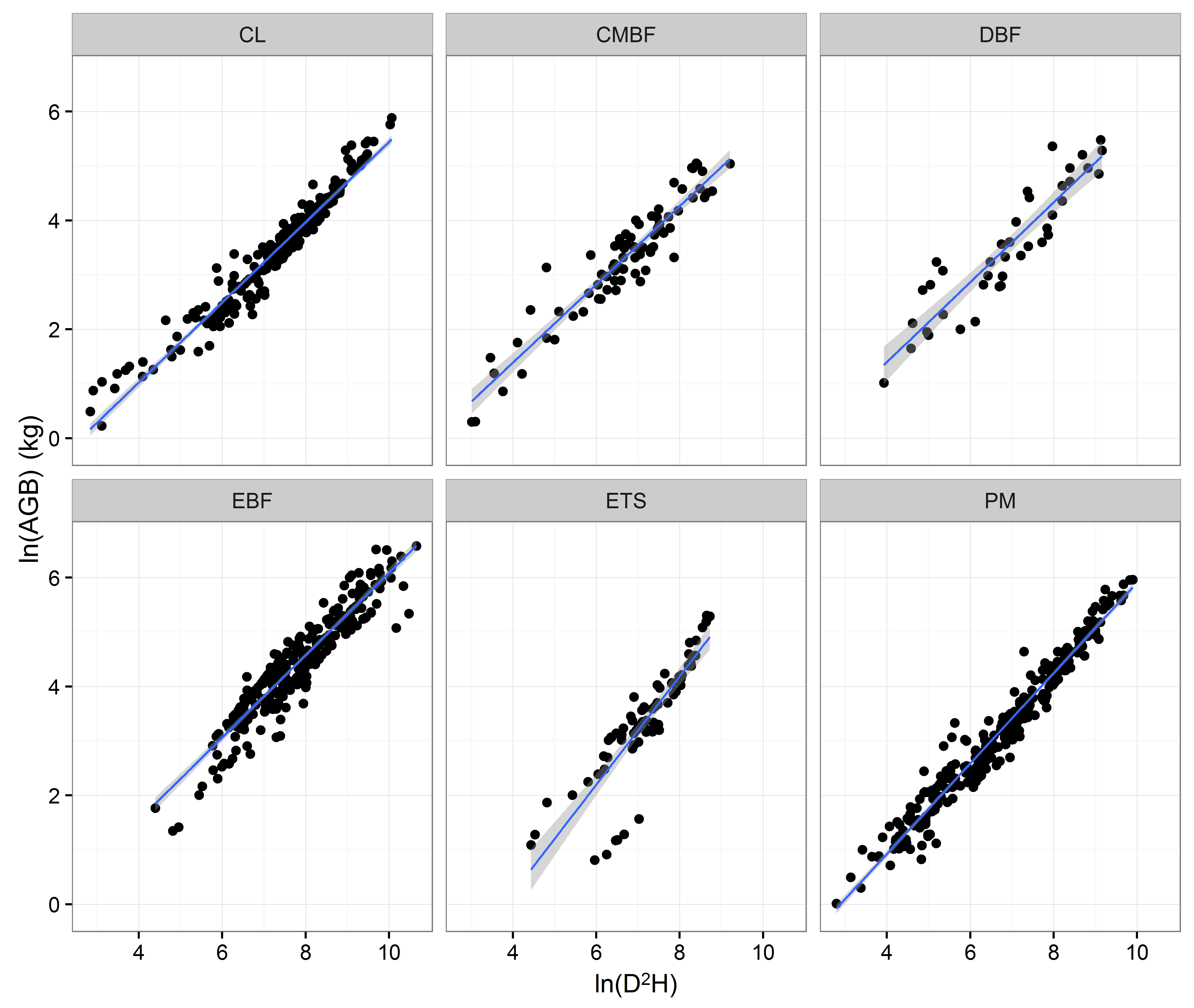
**

CL: *Cunninghamia lanceolata* forest, CMBF: Coniferous and broadleaf mixed forest, DBF: Subtropical deciduous broadleaf forest, EBF: Evergreen broadleaf forest, ETS: *Eucalyptus* tree species forest, and PM: *Pinus massoniana* forest. The rectangular area is the variation range at the 95% confidence interval.
